# Phase II trial of co‐administration of CD19‐ and CD20‐targeted chimeric antigen receptor T cells for relapsed and refractory diffuse large B cell lymphoma

**DOI:** 10.1002/cam4.3259

**Published:** 2020-07-01

**Authors:** Wei Sang, Ming Shi, Jingjing Yang, Jiang Cao, Linyan Xu, Dongmei Yan, Meixue Yao, Hui Liu, Weidong Li, Bing Zhang, Kemeng Sun, Xuguang Song, Cai Sun, Jun Jiao, Yuanyuan Qin, Tingting Sang, Yuanyuan Ma, Mei Wu, Xiang Gao, Hai Cheng, Zhiling Yan, Depeng Li, Haiying Sun, Feng Zhu, Ying Wang, Lingyu Zeng, Zhenyu Li, Junnian Zheng, Kailin Xu

**Affiliations:** ^1^ Department of Hematology the Affiliated Hospital of Xuzhou Medical University Xuzhou China; ^2^ Cancer Institute Xuzhou Medical University Xuzhou China; ^3^ Blood Diseases Institute Xuzhou Medical University Xuzhou China; ^4^ Key Laboratory of Bone Marrow Stem Cell Jiangsu Province China; ^5^ Department of Epidemiology and Biostatistics School of Public Health Xuzhou Medical University Xuzhou China; ^6^ Department of Pathology the Affiliated Hospital of Xuzhou Medical University Xuzhou China; ^7^ Department of Radiology the Affiliated Hospital of Xuzhou Medical University Xuzhou China

**Keywords:** CAR‐T, CD19, CD20, clinical trial, DLBCL

## Abstract

**Purpose:**

Anti‐CD19 chimeric antigen receptor T (CAR‐T) cell therapy has demonstrated remarkable efficacy for refractory and relapsed diffuse large B cell lymphoma (R/R DLBCL). However, this therapy failed in nearly 25% patients mainly due to antigen loss. The authors performed a phase Ⅱ trial by coadministration of anti‐CD19 and anti‐CD20 CAR‐T cells treatment for R/R DLBCL and evaluated its efficacy and toxicity.

**Methods:**

Totally 21 patients with DLBCL were enrolled in this study. The patients were conditioned with fludarabine and cyclophosphamide before the infusion of anti‐CD19 and anti‐CD20 CAR‐T cells. Treatment response, toxicity, and persistence were monitored continuously.

**Results:**

Of the 21 patients received the treatment, the objective response rate (ORR) is 81.0% (95% confidence interval [CI], 58.1%‐94.6%) with four cases of bulk (4/5) and one case of testis involvement; 52.4% (95% CI, 29.8%‐74.3%) had a complete response (CR). Peak levels of anti‐CD19 and anti‐CD20 CAR cells were associated with response (*P* = .007 and .002). Grade 3‐4 cytokine release syndrome (CRS) and neurologic events occurred in 28.5% and 9.5% patients, respectively. Median overall survival (OS) and progression‐free survival (PFS) were 8.1 and 5.0 months, respectively. The maximum standard uptake value (SUVmax) of CD4/CD8 ratio before and after infusion were associated with responses, and the total lesion glycolysis (TLG) before infusion correlates with cytokines level.

**Conclusions:**

Coadministration of anti‐CD19 and CD20 CAR‐T cells therapy for DLBCL is feasible with manageable toxicity. Cytokine markers are related to toxicity and SUVmax could predict efficacy. This trial was registered at www.clinicaltrials.gov as NCT03207178.

## Graphical abstract

In this study, we confirmed that combination of anti‐CD19 and anti‐CD20 CAR‐T cells therapy strategy was efficient (81% ORR and 52.4% CR respectively) for R/R DLBCL, and that the toxicities was transient and manageable. To our knowledge, this is the first report that combination of anti‐CD19 and anti‐CD20 double‐target CAR‐T cell therapy for R/R DLBCL, and our data demonstrated the feasibility of combination of anti‐CD19 and anti‐CD20 CAR‐T cells therapy for R/R DLBCL.

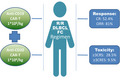

## 1 INTRODUCTION

Diffuse large B cell lymphoma (DLBCL) accounts for approximately 40% of non‐Hodgkin lymphoma (NHL). Due to high heterogeneity, nearly 40% of DLBCL patients did not benefit from rituximab‐based immunochemotherapy.^1^, ^2^ R‐CHOP plus X regimen or rituximab‐based high‐dose regimen also failed to improve overall survival (OS) for some patients, especially those with MYC and BCL‐2/BCL‐6 double hit/expression, activated B cell (ABC), and CD5‐positive DLBCL.^3^, ^4^ In addition, elder patients tend to be more ABC subtype, complexed molecular genetic background, and poor tolerance to chemotherapy, resulting in worse prognosis.^5^, ^6^ For cases of R/R DLBCL, the 3‐year progression‐free survival (PFS) after high‐dose chemotherapy and hematopoietic stem cell transplantation (HSCT) is only 30%‐40% and the median OS is 4.4 months for those frail and unfit for HSCT patients.^7^, ^8^ Although novel drugs have improved the survival of individuals with R/R DLBCL, the objective response rate (ORR) is still less than 40%.^9^, ^10^, ^11^

Chimeric antigen receptor (CAR) T cell therapy targeting CD19 has achieved great success in R/R DLBCL treatment with about 50% CR and a greater than 80% ORR.^12^, ^13^, ^14^ However, since nearly 25% of anti‐CD19 CAR‐T cell therapy for R/R DLBCL failed due to antigen loss,^15^ and CAR‐T cell therapy targeting other targets such as CD20 and CD22 has achieved encouraging results,^16^, ^17^ we speculated that a combined‐target CAR‐T cell treatment to cover more than one target might potentially increase the efficacy by remedying antigen loss‐associated failure. We have previously demonstrated both efficacy and feasibility of combined anti‐CD19 and anti‐BCMA CAR‐T cells for R/R multiple myeloma (ChiCTROIC‐17011272).^18^ These findings prompted us to explore the efficacy and potential adverse effects including CRS and CAR‐T cell therapy‐related encephalopathy syndrome (CRES) in R/R DLBCL when multiple CAR‐T cells were coadministered. Therefore, we treated 21 R/R DLBCL patients with coadministration of anti‐CD19 and anti‐CD20 CAR‐T cells after lymphodepleting and evaluated the safety and efficacy of the dual‐targeted CAR‐T cell therapy.

## 2 MATERIALS AND METHODS

### 2.1 Study design and patients

**FIGURE 1:**
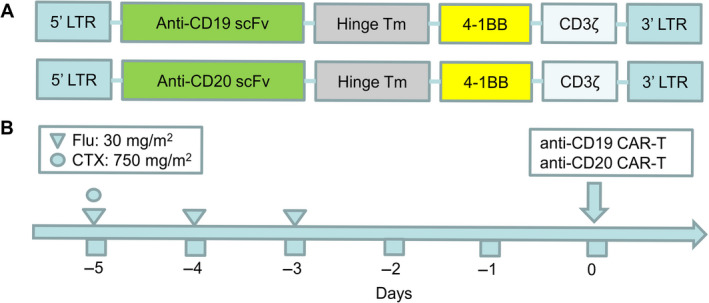
Design of chimeric antigen receptor (CAR) targeting CD19 and CD20. (A) Schematic of anti‐CD19 CAR and anti‐CD20 CAR. Single‐chain (sc) Fv region recognizes CD19 or CD20, CAR contained 4‐1BB costimulatory domain and CD3‐ζ T cell activation domain. (B) Fludarabine combined with cyclophosphamide was used as the pretreatment regimen. Cells were ready for infusion on day 0

This trial was designed by Blood Disease Institute of Xuzhou Medical University with a registration in ClinicalTrials.gov (NCT03207178), and the CAR was assembled in a lentivirus encoding a murine CD19 or CD20‐specific single chain variable fragment combined with the 4‐1BB costimulatory domain and the CD3ζ domain (Figure 1A). The enrolled patients met the following criteria: (a) DLBCL was confirmed by pathology and immunohistochemistry; (b) Patients have previously underwent immunochemotherapy based on anti‐CD20 antibody and anthracycline; (c) At least one measurable lesion was present (refer to the International Working Group [IWG] standard); (d) Nether radiotherapy nor systemic therapy has been administered two weeks before lymphocyte collection; (e) Physical score >50 (Karnofsky Performance Status); and (f) Normal liver, kidney, and cardiac function. Patients were excluded if (a) with a serious autoimmune disease or other tumor disease; (b) participated in other clinical trials of new drugs within the past 3 months; (c) with systemic or local uncontrollable infection; (d) with active hepatitis B and C infection; (e) with HIV infection; and (f) with psychiatric disorders. The CAR‐T cell therapy were conducted between March 2017 and July 2018. The end of follow‐up was October 31, 2018, and the median follow‐up time was 6.6 months (ranged 0.3‐16.4). Clinical trial was approved by the Institutional Review Board of the Affiliated Hospital of Xuzhou Medical University in accordance with Declaration of Helsinki principles. All enrolled patients signed the informed consent prior to the study. Generation of CAR‐T cells, quantitative polymerase chain reaction, image analysis, and cytokine assays are described in the Supplementary Material.

### 2.2 Lymphodepletion chemotherapy and CAR‐T cells infusion

Of the 21 patients, 19 received fludarabine (30 mg/m^2^/d) at days of −5 to −3 and cyclophosphamide (750 mg/m^2^/d) at day −5; and the remaining two patients were treated with ifosfamide (2 g/d) at days of −5 to −3. None of the patients underwent additional chemotherapy as a bridging therapy. The median number of anti‐CD19 CAR‐T cells anti‐CD20 CAR‐T cells was 1.0 × 10^6^/kg (0.2‐4.0 × 10^6^/kg) and 1.0 × 10^6^/kg (0.1‐4.0 × 10^6^/kg), respectively.

### 2.3 Assessment of clinical response and toxicity

The primary end point of the study was ORR. The secondary end point of the study was the CR status at month 6, median OS and PFS, safety, and expansion of CAR‐T cells in vivo. Response of treatment was defined according to the standard international criteria.^19^ For CRS diagnosis and severity assessment, the grading standard were based on that revised criteria by Lee et al.^20^ Other adverse events were evaluated according to the Common Nervous Adverse Events Standards (CTCAE) Version 4.03.^21^

### 2.4 Statistical methods

The study was determined to provide 80% power to test the null hypothesis that the ORR would be 20% or lower vs the alternative hypothesis that it would be 40% or higher. A prespecified rate of response of 20% on the basis of historical values for refractory diffuse large B cell lymphoma.^22^, ^23^, ^24^, ^25^ This design minimized the maximum sample size among all participants in the Simon 2‐stage optimal design with 1‐sided 10% type I error rate. Under the assumption of 5% ineligibility, 21 patients were planned. The 95% CIs for the ORR were calculated using the binomial exact method.

Patients' demographics and clinical characteristics have been summarized using descriptive statistics. PFS was defined as the time from CAR‐T cell infusion until disease progression or death, whichever occurred first. OS was determined as the time from CAR‐T cell infusion until death from any cause. Patients were censored at the date on which they were last known to be alive and/or progression‐free. The median 2‐sided PFS and OS with the 95% CIs were estimated using the Kaplan‐Meier method. For continuous variables that conform to normal distribution, *t* test was used. For those that do not conform to normal distribution, Wilcoxon signed‐rank test was used for paired samples, and Mann‐Whitney *U* test was used for independent samples. *P* values less than .05 were considered significant.

## 3 RESULTS

### 3.1 Patients characteristics

A total of 25 patients with R/R DLBCL were originally enrolled. However, one of them failed to collect sufficient T lymphocytes; CAR‐T cell expansion in vitro failed in two of them, and another patient died due to rapid progressive disease (PD) before CAR‐T cell infusion. Therefore, 21 patients received CAR‐T cell infusion according to the treatment schema (Table 1; Table S1, and Figure 1B). Among them, four patients were MCY/BCL2 double expression, five patients were with bulky mass (≥7.5 cm), and one patient was with MCY/BCL2 rearrangement, one CD5 positive patient had testicular involvement, and one patient received autologous HSCT before CAR‐T cell therapy. Fourteen patients were immunochemotherapy refractory as defined as the best response was stable disease (SD) or PD after two cycles of a standard or conventional first‐line treatment regimen, or failed to achieve CR after two cycles. Seven patients met the criteria of relapse that CR was achieved after treatment, but relapse occurred within 1 year after treatment. Patients received a median of 3‐line (range, 1‐6) regimens before protocol enrollment (Table S2).

**TABLE 1:** Characteristics of the patients at baseline

Characteristic	Patients, n (%)
Total patients	21 (100%)
Median age, years (range)	55 (23‐72)
Sex	
Male	13 (61.9%)
Female	8 (38.1%)
Disease stage	
I‐II	3 (14.3%)
III‐IV	18 (85.7%)
NCCN‐IPI group	
Low‐intermediate: 2‐3	6 (28.6%)
High‐intermediate: 4‐5	13 (61.9%)
High: ≥6	2 (9.5%)
Extranodal disease	6 (28.6%)
BM involved	5 (23.8%)
CNS involved	3 (14.3%)
Bulky	5 (23.8%)
Cell of origin	
GCB	16 (76.2%)
NON‐GCB	5 (23.8%)
MCY/BCL2 DE	4 (19.0%)
Refractory DLBCL	15 (71.4%)
Median previous therapies (range)	3 (1‐6)
Conditioning therapy	
FC	19 (90.5%)
Others	2 (9.5%)

Abbreviations: BM, bone marrow; CNS, central nervous system; DE, double expression; FC, fludarabine and cyclophosphamide; GCB, germinal center B cell; NCCN‐IPI, National Comprehensive Cancer Network‐International Prognostic Index.

### 3.2 Response assessment

**FIGURE 2:**
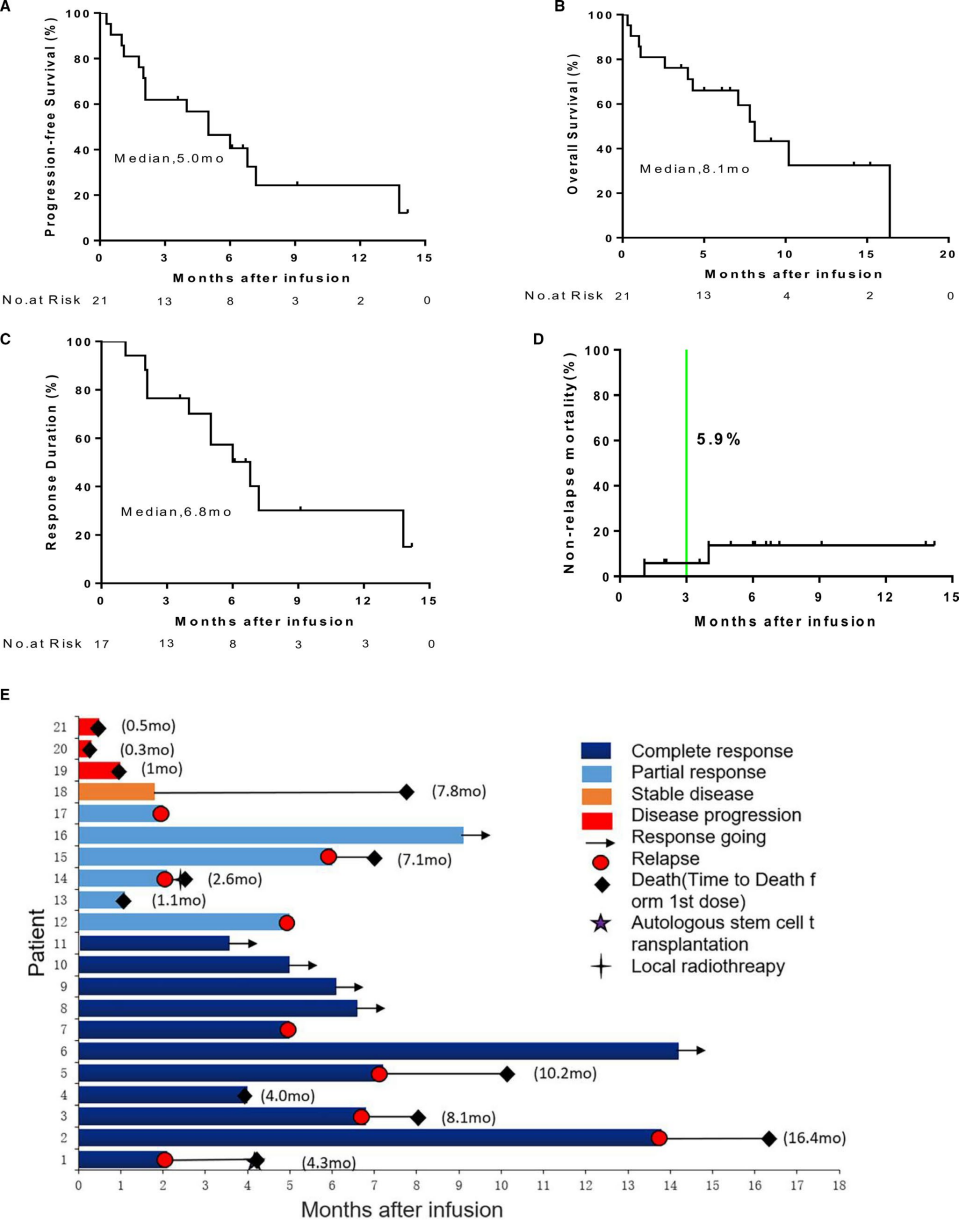
Function of anti‐CD19 and anti‐CD20 CAR‐T cells in R/R DLBCL. (A, B) The median PFS and OS of 21 patients are shown. (C) The response duration and non‐relapse mortality (NRM) (D) of 17 patients with response are shown. The survival fractions were calculated by the Kaplan‐Meier method, and the (green) lines indicate censored patients. (E) The clinical responds and survival condition of anti‐CD19 combined with anti‐CD20 CAR‐T cells therapy

The median time between recruitment and infusion was 32 days (range, 16‐45). The ORR and CR rates at 3 months were 81.0% (17 patients, 95% CI, 58.1%‐94.6%) and 52.4% (11 patients), and the 6‐month sustained ORR and CR rates were 46.2% (6 patients, 95% CI, 19.2%‐74.9%) and 40.0% (4 patients, 95% CI, 12.2%‐73.84%), respectively. The median PFS, OS, and duration of response were 5.0 months (95% CI, 2.3‐7.7), 8.1 months (95% CI, 6.5‐9.6), and 6.8 months, respectively (Figure 2A‐C). Of the five patients with bulky mass, four patients responded, and two of them achieved CR (Table 2). Of the four patients with MYC/BCL‐2 expression, three achieved CR, and one achieved PR. The patient of MYC/BCL2 rearrangement developed PD. CR was achieved in a CD5 positive case with testicular involvement. There were three patients with central nervous system (CNS) involvement, including two of primary and one secondary cases. Of the two patients with primary CNS lymphoma, one achieved PR (P12) and the other showed SD (P18). The patient with secondary CNS involvement (P13) achieved PR at 3 months. At the end of follow‐up, 10 patients died due to PD. Two patients died without relapse, one was due to pulmonary infection at day 42 and the other suffered from severe intestinal infection at day 121 after CAR‐T cell infusion (Figure 2D,E).

**TABLE 2:** Baseline characteristics of patients with bulky

No.	Age (y)	Sex	Stage/status	Previous therapies	Site involved	Response	The longest diameter (cm)	
							Before	After
1	58	F	ⅣB/PD	3	Abdomen	CR	11.0	1.3
2	70	M	ⅣA/PD	3	Abdomen	CR	8.0	1.2
3	48	M	ⅣA/PD	5	Neck (L)	PR	11.9	5.1
4	46	F	ⅣA/PD	3	Abdomen	PR	7.8	3.2
5	57	M	ⅢA/PD	1	Iliac fossa (L)	PD	9.1	9.3

Abbreviations: CR, complete remission; F, female; L, left; M, male; PD, progressive disease; PR, partial remission.

### 3.3 Toxicities of CAR‐T cell

The adverse events were detailed in Table 3. CRS occurred in all 21 patients with 6 (28.5%) of them as grade 3‐4 CRS. The median time from CAR‐T cell infusion to CRS occurrence was 2 days (ranged 0‐5) and the median CRS duration was 5 days (ranged 2‐14). Four patients with CRS returned to normal after dexamethasone treatment and none of them needed tocilizumab. Five patients were with CRES, and the median duration was 5 days (ranged 3‐8). For the two patients with grade 3‐4 CRES, the duration were 7 and 8 days, respectively. The main symptoms of CRES were dysphoria (n = 3) and delirium (n = 2) with a median duration of 3 days (ranged 2‐7). Of the five patients over 60 years old, one had grade four CRS and another had CRES.

**TABLE 3:** Treatment‐emergent adverse events

Adverse events	No. of patients, (%, n = 21)	
	All grades	Grade ≧3
Fatigue	16 (76.2%)	5 (23.8%)
Dyspnea	7 (33.3%)	3 (14.3%)
Nausea	7 (33.3%)	0
Anorexia	11 (52.4%)	3 (14.3%)
Diarrhea	2 (9.5%)	0
Vomit	5 (23.8%)	0
Constipation	4 (19.0%)	0
Acute kidney injury	2 (9.5%)	0
Pulmonary infection	4 (19.0%)	1 (4.8%)
^a^CRS, specific symptoms		
Fever	20 (95.2%)	2 (9.5%)
Rigors	6 (28.6%)	0
Hypotension	10 (47.6%)	4 (19.0%)
Hypoxia	10 (47.6%)	3 (14.3%)
Tachycardia	13 (61.9%)	4 (19.0%)
Neurotoxicity, specific symptoms		
Encephalopathy	5 (23.8%)	2 (9.5%)
Delirium	2 (9.5%)	2 (9.5%)
Somnolence	1 (4.8%)	0
Restlessness	3 (14.3%)	2 (9.5%)
Dysmnesia	1 (4.8%)	0
Tremor	1 (4.8%)	0
Hematologic events		
Leukopenia	16 (76.2%)	10 (47.6%)
Neutropenia	16 (76.2%)	11 (52.4%)
Anemia	17 (81.0%)	6 (28.6%)
Thrombocytopenia	6 (28.6%)	6 (28.6%)
Laboratory abnormalities		
ALT elevation	4 (19.0%)	0
AST elevation	4 (19.0%)	0
Hyperuricemia	3 (14.3%)	0
Hypoalbuminemia	7 (33.3%)	3 (14.3%)
Hypokalemia	8 (38.1%)	2 (9.5%)
Hyponatremia	6 (28.6%)	0
Hypocalcemia	4 (19.0%)	0

Abbreviations: ALT, alanine aminotransferase; AST, aspartate aminotransferase; LDH, lactate dehydrogenase.

^a^ CRS was graded per a modified grading system proposed by Lee et al.^20^

### 3.4 Cytokine and laboratory assessment

**FIGURE 3:**
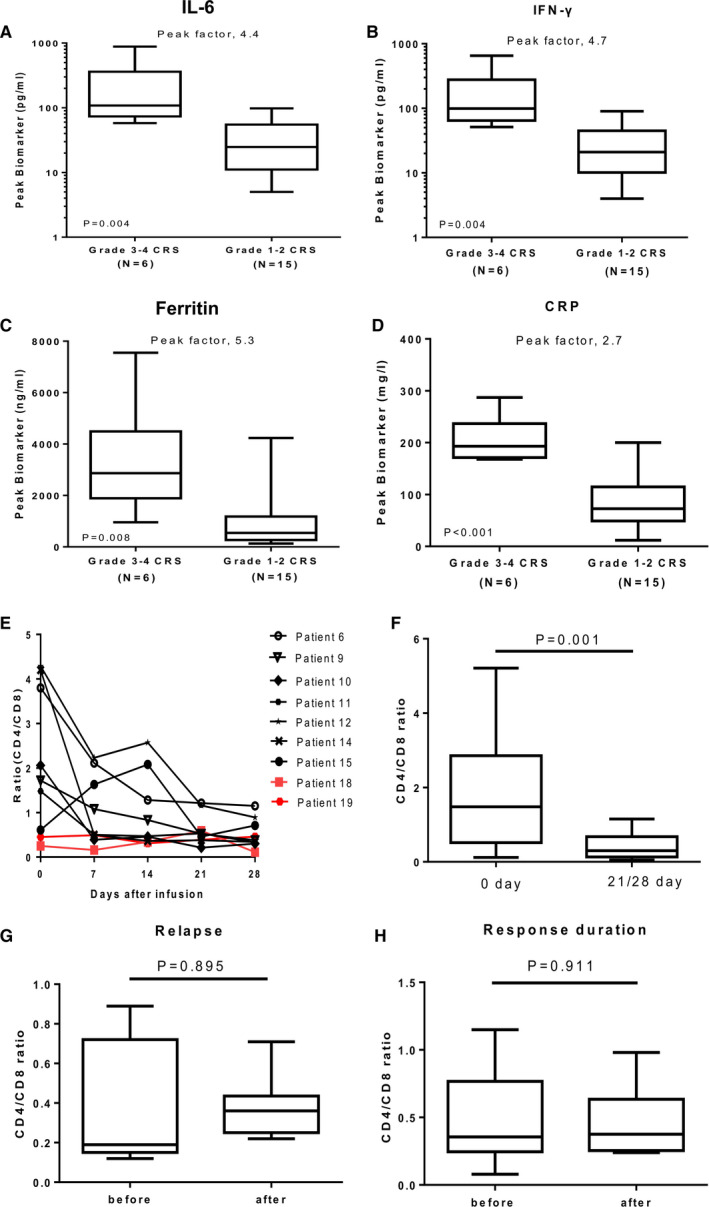
Changes of serum biomarkers and CD4/CD8 ratio after CAR‐T cell infusion. (A‐D) IL‐6, IFN‐γ, ferritin, and CRP were associated only with CRS. The peak value is defined as the maximum level of the cytokine after baseline within a month of cell infusion. The peak factor is the value in patients with CRS of grade 3‐4 vs those with events of grade 1‐2. The horizontal line within each box represents the median, the lower and upper borders of each box represent the 25th and the 75th percentiles, respectively, and the I bars represent the minimum and maximum range. The Mann‐Whitney *U* test or *t* test was used for statistical analysis. (E) Dynamic changes of CD4/CD8 ratio in patients after CAR‐T cell infusion. P6, 9, 10, and 11 were CR patients, P12, 14, and 15 were PR patients, P18 was SD patient, and P19 was PD patient. (F) Dynamic changes of CD4/CD8 ratio in 17 patients with response. (G) Dynamic changes of CD4/CD8 ratio in 9/17 patients with relapse after CAR‐T cell infusion. (H) Dynamic changes of CD4/CD8 ratio in patients with response duration. The Wilcoxon rank‐sum test or *t* test were used for statistical analysis

The levels of IL‐6, ferritin, C‐reactive protein (CRP), and IFN‐γ reached their peak between days of 7 and 14 after CAR‐T cell infusion. The peak levels of these factors in patients with grade 1‐2 CRS (n = 15) were significantly lower than those with grade 3‐4 CRS (n = 6) (Figure 3A‐D). Hematologic toxicities were found in 17 patients, including 16 cases of neutropenia (76.2%), 17 cases of anemia (81.0%), 6 cases of thrombocytopenia (28.6%), and 16 cases with a decreased white blood cell count (76.2%). Grade 3 hematologic toxicities of neutropenia, anemia, and thrombocytopenia were 52.4%, 28.6%, and 28.6%, respectively. Eleven patients received an injection of recombinant human granulocyte macrophage‐stimulating factor to increase leukocyte levels, four and three patients required platelet and red blood cell transfusion, respectively.

### 3.5 Assessment of B cells and immunoglobulin

B cells and immunoglobulin were measured to assess the immune status of B cells after CAR‐T cell therapy (data not shown). In five (23.8%) patients, B cells were not detected in peripheral blood before CAR‐T cell infusion, including four patients with response and one patient without response eventually. Two weeks after CAR‐T cell infusion, B cells were undetectable in 11 responsive patients and one nonresponsive patient; and detectable in two non‐responsive patients. B cells recurred in five of nine relapsed patients, and the median time is 6.1 months (ranged 4‐13.7) after CAR‐T cell infusion. Of the 17 patients with response, 14 (82.4%) showed a progressive reduction of serum immunoglobulin levels one week after infusion. Eight of the 21 (38.1%) patients received intravenous immunoglobulin during CAR‐T cell therapy.

### 3.6 Assessment of T cells and CD4/CD8 ratio

T cells in the peripheral blood of patients were measured to assess cellular immune status after CAR‐T cell therapy (data not shown). A dynamic reduction of CD4/CD8 ratio occurred in 15 of 17 responsive patients. Among the four nonresponsive patients, the ratio did not change in three and declined in one (Figure 3E). The CD4/CD8 ratio in the 17 responsive patients at 4 weeks after CAR‐T cell infusion was significantly lower than that before infusion (Figure 3F, *P* = .001). Of the 17 responsive patients, nine relapsed later but their CD4/CD8 ratio displayed no change before and after the relapse (Figure 3G, *P* = .895). Of note, the CD4/CD8 ratio in the six patients with continuous response did not change even by the end of the follow‐up (Figure 3H, *P* = .911).

### 3.7 In vivo expansion and persistence of CAR‐T cells

**FIGURE 4:**
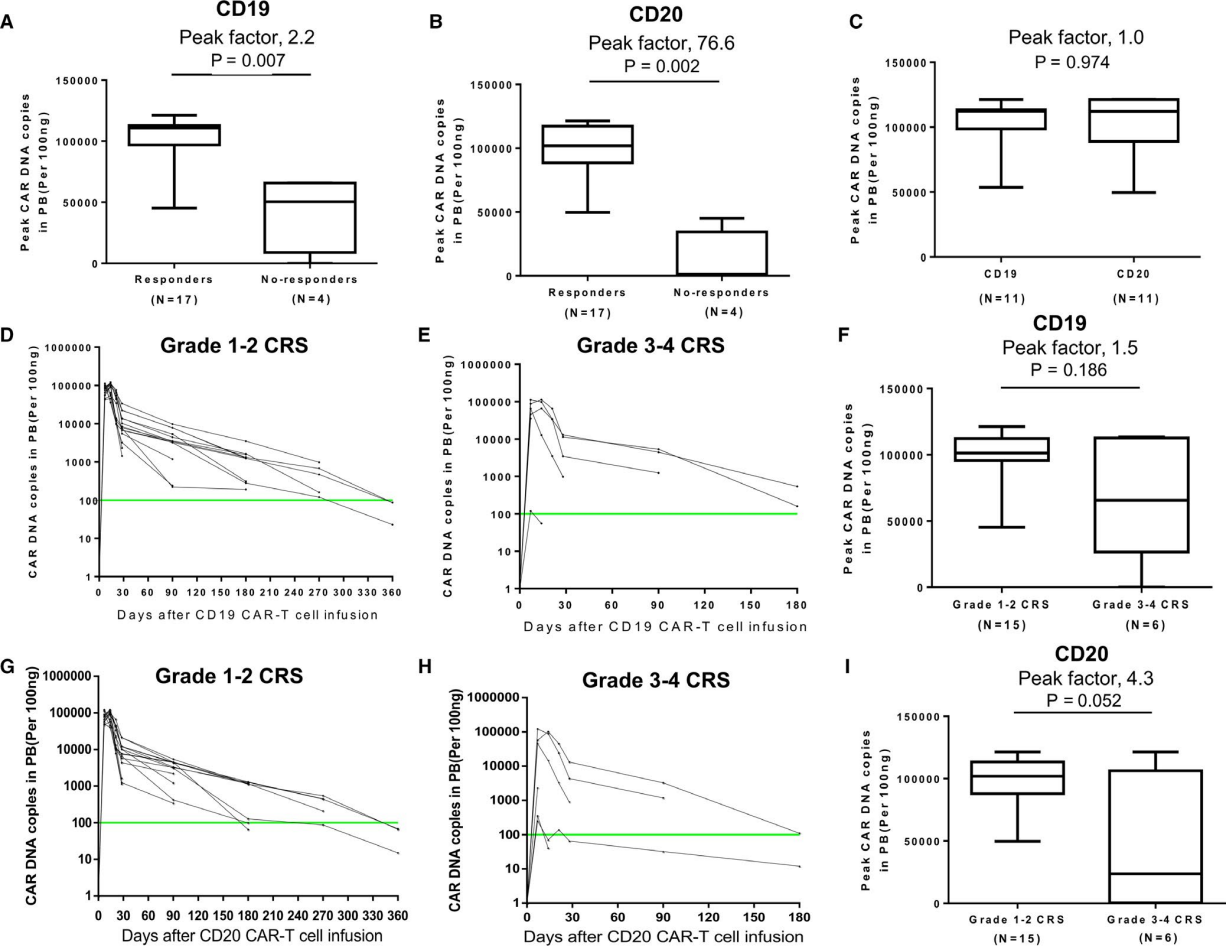
Expansion and persistence of anti‐CD19 and anti‐CD20 CAR‐T cells in vivo. (A, B) Peak CAR DNA copies of anti‐CD19 and anti‐CD20 CAR‐T cells in the groups with response and without response. (C) Peak CAR DNA copies of anti‐CD19 CAR‐T cell and anti‐CD20 CAR‐T cell in patients of CR. (D, E) CAR DNA copies of anti‐CD19 CAR‐T cell at serial time points after infusion in patients who developed grade 1‐2 CRS and those who developed grade 3‐4 CRS. (F) Peak CAR DNA copies of anti‐CD19 CAR‐T cell in the groups with grade 1‐2 and grade 3‐4 CRS. (G, H) CAR DNA copies of anti‐CD20 CAR‐T cell at serial time points after infusion in patients who developed grade 1‐2 CRS and those who developed grade 3‐4 CRS. (I) Peak CAR DNA copies of anti‐CD20 CAR‐T cell in the groups with grade 1‐2 and grade 3‐4 CRS. The horizontal line at 100 copies per microgram of DNA represents the lower limit of quantification of this assay. The Mann‐Whitney *U* test was used for statistical analysis

Significant CAR‐T cell expansion occurred in 17 responsive patients and both anti‐CD19 and anti‐CD20 CAR peaked between 7‐14 days after CAR‐T cell infusion. Of the four non‐responders, three had anti‐CD19 CAR‐T expansion, two had anti‐CD20 CAR‐T expansion, and one amplified neither of them. The expansion of anti‐CD19/CD20 CAR‐T cells in patients with response (n = 17) was significantly higher than those without response (n = 4) (Figure 4A,B, *P* = .007 and .002). For the 11 patients achieving CR, there was no difference between the peaks of anti‐CD19 and anti‐CD20 CAR‐T cells (Figure 4C). In the six patients with grade 3‐4 CRS, the median copies of anti‐CD19 and anti‐CD20 CAR were 65 680 and 23 715 copies/100 ng total DNA, respectively, which were not significantly different from those with grade 1‐2 CRS (n = 15) (Figure 4D‐I). CAR‐T cells could not be detected in the patients after relapse and the two patients with continuous remission, respectively, at 6 and 9 months after CAR‐T cell infusion.

### 3.8 Impact of SUVmax and TLG on response, CRS and CAR‐T cell expansion

**FIGURE 5:**
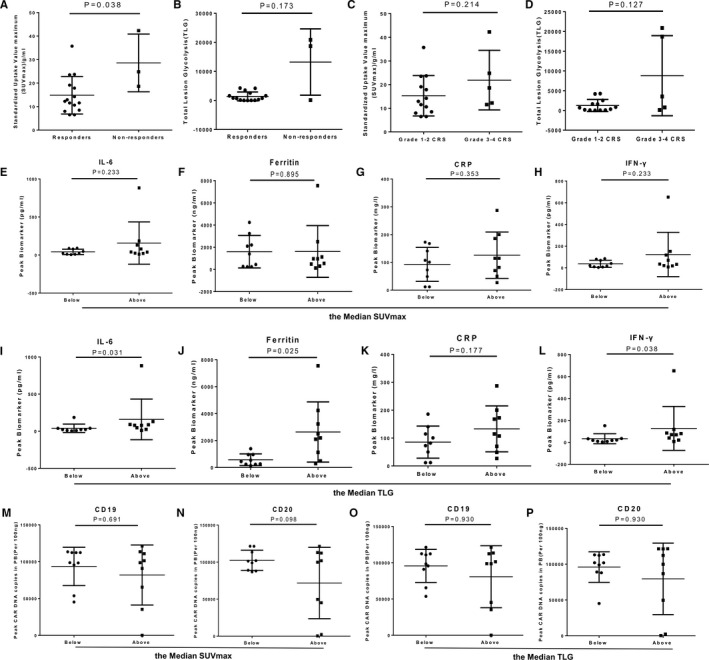
The values of SUVmax and TLG in response, CRS and CAR‐T cells expansion. (A, B) The vaule of SUVmax and TLG in the groups with and without response. (C, D) The vaule of SUVmax and TLG in grade 1‐2 and grade 3‐4 CRS. (E‐H) Peak CAR DNA copies of anti‐CD19 and anti‐CD20 in below and above the median SUVmax/TLG groups. (I‐P) The peak value of serum biomarkers (IL‐6, IFN‐γ, ferritin, and CRP) in below and above the median SUVmax/TLG groups. The peak value is defined as the maximum level of the cytokine after baseline within a month. The data represent the means ± SD. The Mann‐Whitney *U* test or *t* test were used for statistical analysis

Based on PET‐CT, we evaluated the SUVmax and TLG before CAR‐T cell therapy. The SUVmax (g/ml) of 15 evaluable patients with treatment response (median of 12.23, range: 6.49‐35.71) was significantly lower than that of three patients without response (median of 24.8, range: 18.6‐42.29) (Figure 5A, *P* = .038), but the SUVmax is comparable in the six patients with durable remission and the other nine cases (*P* = .541). There was no significant difference in TLG values between responders and non‐responders (Figure 5B). The SUVmax and TLG of evaluable patients with grade 1‐2 CRS (n = 13) were not significantly different from those with grade 3‐4 CRS (n = 5) (Figure 5C,D). SUVmax and cytokine levels were not correlated (Figure 5E‐H). However, high levels of TLG correlate with the levels of IL‐6, ferritin, and IFN‐γ but not the CRP level (Figure 5I‐L). In addition, we evaluated the correlations of SUVmax and TLG with the expansion of CAR‐T cells in vivo. There was no significant difference in the anti‐CD19 CAR‐T and anti‐CD20 CAR‐T expansion between patients whose SUVmax or TLG was over the median and those below (Figure 5M‐P).

## 4 DISCUSSION

To our knowledge, this is the first reported clinical trial of coadministration of anti‐CD19 and anti‐CD20 CAR‐T cell therapy for R/R DLBCL. Although anti‐CD19 CAR‐T cell therapy is effective for R/R DLBCL,^12^, ^13^, ^14^ about 50%‐60% patients relapse after CAR‐T treatment with as high as 27% are antigen loss‐associated.^15^ Based on the expectation that multi‐target CAR‐T cell therapeutic strategy should improve efficacy and potentially solve the problem with antigen loss‐associated failure, we treated R/R DLBCL with the combination of anti‐CD19 and anti‐CD20 CAR‐T cells and achieved a promising response with CR rate of 52.4% and ORR rate of 81% albeit the PFS and DOR were not as encouraging. This could be the results of immune escaping, lacking patient‐originated memory CAR‐T cells, or failure of continued CAR‐T cell expansion. Generally, MYC and BCL‐2/BCL‐6 double‐expression/rearrangement and CD5‐positive DLBCL are resistant to rituximab‐based immunochemotherapy. We noticed in this trail that three out of four patients with MYC/BCL‐2 double expression achieved CR. Due to the limited number of cases, we cannot make solid conclusion that co‐administration of anti‐CD19 and anti‐CD20 CAR‐T cells can overcome the adverse prognosis caused by double expression. In addition to that multiple cases with bulk achieved 80% ORR, we have also noticed that in one patient of CD5‐positive DLBCL with testicular involvement also achieved CR, suggesting that anti‐CD19 and anti‐CD20 CAR‐T cells are capable of passing the blood‐testis barrier.

The universal application of CAR‐T cell therapy is limited mainly due to the complications of CAR‐T cell therapy, especially for ≥grade 3 CRS and CRES.^26^, ^27^ In our trial, 23.8% and 9.5% patients had CRS (≥grade 3) and CRES (≥grade 3), respectively, four patients received emergent glucocorticoid therapy due to hemorheological instability, but none of the patients received tocilizumab. Interestingly, of the 21 patients receiving CAR‐T cells, six elderly achieved 83% ORR with only one of them had grade 3 CRS. More importantly, the toxicity was transient and manageable while the median dose of 1.0 × 10^6^/kg CAR‐T cells were administered and the levels of cytokine peaked 7‐14 days after CAR‐T cell infusion. We have also noticed that the peak levels of the inflammatory cytokines in patients with grade 1‐2 CRS were significantly lower than those with grade 3 CRS and these notifications were consistent with the reports in the literature.^12^, ^13^, ^14^ The hematologic toxicities primarily manifested as manageable hemocytopenia and none of the patients died due to CAR‐T cell treatment‐associated hematologic complications although off‐target effects were noticeable. All responders and a few nonresponders lost B cells two weeks after CAR‐T cell infusion. However, B cells recurred in five of nine relapsed patients with the median time of 6.1 months post CAR‐T cell infusion. Consistently, immunoglobulin levels reduced progressively in the responders and some of the nonresponders and 38.1% patients received intravenous immunoglobulin infusion during CAR‐T cell therapy but none of them had serious infection.

Great expansion of CD8 positive CAR‐T cells was closely related to efficacy, and IL‐2 produced by CD4 positive T cells was important in sustaining its response.^28^ As reported by Turtle and his colleagues, CAR‐T cell products in a 1:1 CD4/CD8 ratio was supposed to be worthy of recommendation.^29^ However, the correlation of CD4/CD8 ratio after CAR‐T cell infusion and efficacy is not clear. As shown in this study, the CD4/CD8 ratio in 15 of 17 responders decreased gradually, whereas the ratios did not change in three of the four nonresponders. So, we propose that the CD4/CD8 ratio could serve as a predictor for CAR‐T cell therapy. We also analyzed the expansion and persistence of CAR‐T cells in patients. We found that all responders exhibited expansion with the peak values of anti‐CD19 and CD20 CAR‐T cells in responders significantly higher than that of the nonresponders. However, there is no significant difference between the peak values of anti‐CD19 and anti‐CD20 CAR‐T cells in the CR patients.

PET‐CT is of great value in assessment of tumor burden and treatment efficacy.^30^ To evaluate the role of metabolic activity of lymphoma on CAR‐T therapy, we analyzed the correlation of SUVmax, and we also evaluated the correlation of TLG, which was determined by SUVmean and TMV. We found that lower SUVmax correlates with better response suggesting high levels SUVmax predict high risk of CAR‐T cells treatment. However, the levels of TLG have nothing to do with efficacy but highly associated with IFN‐γ, IL‐6, and the ferritin levels. Nevertheless, neither SUVmax nor TLG correlate with the occurrence of CRS or CAR‐T cell expansion.

In summary, this trial demonstrated that coadministration of anti‐CD19 and anti‐CD20 CAR‐T cell therapy is safe and feasible with manageable toxicity even for those with bulk, MYC/BCL‐2 double expression, and CD5 positive DLBCL. We also found that CAR‐T cells are capable of passing the blood‐brain and blood‐testis barriers. Finally, reduced CD4/CD8 ratio and lower SUVmax predict a better treatment efficacy.

## CONFLICT OF INTEREST

No potential conflict of interest was disclosed.

## AUTHORS CONTRIBUTIONS

Conception and design: Kailin Xu, Wei Sang, Zhenyu Li, Junnian Zheng. Development of methodology: Kailin Xu, Wei Sang, Jiang Cao, Ming Shi. Acquisition of data (provided animals, acquired and managed patients, provide facilities, etc): Kailin Xu, Wei Sang, Ming Shi, Jingjing Yang, Jiang Cao, Linyan Xu, Dongmei Yan, Meixue Yao, Hui Liu, Hai Cheng, Zhiling Yan, Depeng Li, Haiying Sun, Feng Zhu, Ying Wang. Analysis and interpretation of data (eg, statistical analysis, biostatistics, computational analysis): Wei Sang, Jingjing Yang, Bing Zhang, Kemeng Sun, Xuguang Song, Cai Sun, Jun Jiao, Tingting Sang, Xiang Gao, Lingyu Zeng. Writing, review, and/or revision of the manuscript: All authors. Administrative, technical, or material support (ie, reporting or organizing data, constructing databases): Kailin Xu, Wei Sang, Jiang Cao, Zhenyu Li, Junnian Zheng.

## Supporting information

Table S1‐S2

Supplementary Material

## Data Availability

The data will be provided upon the request.
