# Supplementary material for: Phase II trial of co‐administration of CD19‐ and CD20‐targeted chimeric antigen receptor T cells for relapsed and refractory diffuse large B cell lymphoma
Source: Cancer Med. 2020 Jul 1;9(16):5827–38. doi: 10.1002/cam4.3259 (PMC7433814; doi:10.1002/cam4.3259)
Supplement: Supplementary file 1 — Table S1‐S2 [file CAM4-9-5827-s001.doc]

**Supplementary tables and figures**

**Supplementary Table 1. Supplementary characteristics of patients**

| Patient | Age  (yr) | Sex | Stage | NCCN-  IPI score | Extranodal involved | BM involved | CNS involved | Bulky | GCB | Status | Conditioning |
| --- | --- | --- | --- | --- | --- | --- | --- | --- | --- | --- | --- |
| 1 | 57 | M | Ⅳ | 5 | N | N | N | N | Y | Ref | IFO |
| 2 | 61 | F | Ⅳ | 4 | N | N | N | N | Y | Ref | FC |
| 3 | 58 | F | Ⅳ | 5 | N | N | N | Y | Y | Ref | FC |
| 4 | 55 | M | Ⅳ | 4 | N | N | N | N | N | Rel | IFO |
| 5 | 67 | M | III | 4 | N | N | N | N | Y | Ref | FC |
| 6 | 70 | M | Ⅳ | 4 | N | Y | N | Y | Y | Ref | FC |
| 7 | 28 | M | Ⅳ | 5 | N | Y | N | N | Y | Ref | FC |
| 8 | 41 | F | III | 2 | N | N | N | N | N | Rel | FC |
| 9 | 54 | F | III | 4 | N | N | N | N | Y | Ref | FC |
| 10 | 45 | F | Ⅳ | 3 | N | N | N | N | Y | Ref | FC |
| 11 | 72 | F | III | 3 | N | N | N | N | Y | Ref | FC |
| 12 | 43 | M | Ⅰ | 3 | Central | N | Y | N | Y | Rel | FC |
| 13 | 46 | F | Ⅳ | 6 | N | Y | Y | Y | Y | Ref | FC |
| 14 | 23 | M | Ⅱ | 2 | Gastric | N | N | N | Y | Ref | FC |
| 15 | 48 | M | Ⅳ | 5 | N | N | N | Y | N | Ref | FC |
| 16 | 62 | F | III | 4 | N | N | N | N | N | Ref | FC |
| 17 | 43 | M | IV | 5 | N | Y | N | N | Y | Rel | FC |
| 18 | 24 | M | I | 3 | Central | N | Y | N | Y | Rel | FC |
| 19 | 57 | M | III | 4 | Adrenal gland | N | N | Y | Y | Rel | FC |
| 20 | 67 | M | Ⅳ | 7 | Gastric | N | N | N | N | Ref | FC |
| 21 | 57 | M | Ⅳ | 5 | Gastric | Y | N | N | Y | Ref | FC |

Abbreviations: NCCN-IPI, National Comprehensive Cancer Network-International Prognostic Index; BM, bone marrow; CNS, central nervous system; GCB, germinal center B cell; M, man; F, female; Y, yes; N, no. Ref, refractory; Rel, relapsed; IFO, Ifosfamide; FC, fludarabine and cyclophosphamide;

**Supplementary Table 2. Therapies received before CAR-T protocol enrollment**

| patient | treatment |
| --- | --- |
| 1 | -RCHOP/-R-EPOCH |
| 2 | -CHOP/-EPOCH |
| 3 | -R-CHOP/-CHOP/-GDP |
| 4 | -R-CHOP |
| 5 | -R-CHOP/-E-CHOP/-GDP/-MINE |
| 6 | -COEP/-R-EPOCH/-R-DICE |
| 7 | -BETD/-CETD/-VP16/-R-GP/-R-ID/-RTX to cervical region |
| 8 | -CHOP/-R |
| 9 | -R-CHOP/-R-GDP |
| 10 | -RTX to to cervical region/-CVP/-CHOP/-R-GDP |
| 11 | -COP/-CHOP |
| 12 | -RTX to brain/-R+MTX/-R+hyper-CVAD B/-Hyper-CVAD B/-MT /-MTD/-MT |
| 13 | -VP/-R-VDP/-R-Gemox |
| 14 | -R-CHOP/-R-DHAP/-ICE |
| 15 | -R-CHOP/-CVAD/-R-MINE/-COPE/-ESHAP |
| 16 | -R-COP/-R-GDP |
| 17 | -R-ESHAP/-R-GDP/-CHOP |
| 18 | -R-MA/Auto-SCT/-R-TDT |
| 19 | -CHOPE |
| 20 | -R-CHOP/-RTX to Lumbar/-R-COP/-R |
| 21 | -COP/-CHOP/-Gemox/-R-DA-EPOCH/-R-MINE |

Abbreviations: BETD：bleomycin, etoposide, epirubicin, dacarbazine; CETD：cyclophosphamide, etoposide, epirubicin, dacarbazine; CHOP: cyclophosphamide, vincristine, epirubicin, prednisone; COP: cyclophosphamide, vincristine, prednisone; CHOPE: cyclophosphamide, vincristine, epirubicin, prednisone, etoposide; CVAD: cyclophosphamide, vincristine, epirubicin, dexamethasone; DHAP: Cisplatin, cytarabine, dexamethasone; DICE: dexamethasone, ifosfamide, Cisplatin, etoposide; DXM: dexamethasone; EPOCH: etoposide, cyclophosphamide, vincristine, epirubicin, prednisone; ESHAP: etoposide, cytarabine, Cisplatin, dexamethasone; GDP: gemcitabine, cisplatin, dexamethasone; Gemox: gemcitabine, oxaliplatin; HDT+Auto-SCT: High dose therapy and autologous stem cell transplantation; HD-MTX: High dose methotrexate; Hyper-CVAD B: methotrexate, cytarabine; ICE: ifosfamide, cisplatin, etoposide; MINE: mesna, ifosfamide, mitoxantrone, etoposide; MT: high-dose methotrexate, temozolomide; MTD: high-dose methotrexate, dexamethasone, temozolomide; MTX: methotrexate; R: rituximab; R-DA-EPOCH: Dose-adjusted rituximab, etoposide, cyclophosphamide, vincristine, epirubicin, prednisone; R-MA: rituximab, mitoxantrone, cytarabine; R-GP: rituximab, gemcitabine, cisplatin; R-ID: rituximab, ifosfamide, dexamethasone; R-TDT: rituximab, dexamethasone, lenalidomide, temozolomide; RTX: radiation therapy; VDP: vincristine, daunorubicin, prednisone; VP: vincristine, prednison; VP 16: etoposide.
