# Supplementary material for: Phase II trial of co‐administration of CD19‐ and CD20‐targeted chimeric antigen receptor T cells for relapsed and refractory diffuse large B cell lymphoma
Source: Cancer Med. 2020 Jul 1;9(16):5827–38. doi: 10.1002/cam4.3259 (PMC7433814; doi:10.1002/cam4.3259)
Supplement: Supplementary file 2 — Supplementary Material [file CAM4-9-5827-s002.doc]

Supplemental materials and methods

*Generation of CAR-T cells and Ex Vivo Assays*

T cells were isolated by EasySep Human T Cell Isolation Kit (Stem cell), and cultured with X-vivo 15 (Lonza) containing 10% fetal bovine serum (FBS)(Gibco), 500 IU recombinant human interleukin-2 (Beijing Four Rings Biopharmaceutical.) and 4 ng/µl recombinant human interleukin-21(Biolegend). T cells was adjusted to 1 × 10^6^/ml and seeded in a T flask coated with anti-mouse Fc antibody (Jackson Immunoresearch), then activated with anti-CD3 and anti-CD28 monoclonal antibodies (Novoprotein) at a final concentration of 0.25 µg/ml and 1 µg/ml, respectively. After 24 hours of culture, the cell density was adjusted to 2 × 10^6^/ml, and the cells were mixed with lentiviruses with a titer of 2 × 10^7^/ml (MOI = 10) at a volume ratio of 1: 1. The cells were incubated in a CO_2_ incubator for 24 hours, and the density was adjusted to 1 × 10^6^/ml after the medium was replaced with fresh medium. Cell viability was determined using an Annexin V-FITC/PI apoptosis assay kit (Multisciences Biotech). The proportion of CAR-positive cells was determined using anti-scFv primary antibody and APC secondary antibody (Yang’s lab, Shanghai Jiao Tong University). The cytotoxic activity of CAR-T cells was demonstrated via in vitro killing of Raji cells (Yang’s lab, Shanghai Jiao Tong University) by the CAR-T cells. BacT-ALERT 3D120 (bioMerieux) was used for detection of the bacterial culture of the final product. The endotoxin level of the cells was determined (≤0.25 EU/mL) using a limulus lysate test. The total volume of the final product was 100mL. Quantitative polymerase chain reaction, image analysis and cytokine assays are described in the supplementary material.

*Measurement of CAR-T cells expansion and persistence*

After infusion of CAR-T cells, mononuclear cells from peripheral blood samples were collected weekly. Genomic DNA was extracted using a TIANamp Genomic DNA Kit (TIANGEN), and the CAR gene was detected via quantitative polymerase chain reaction (qPCR). We selected the scFv fragment as the amplification region (Anti-CD19-CAR: forward primer GAGTCACCATCAGTTGCAGG, reverse primer TCTTCTTGCTCCAGGTTGCT; Anti-CD20-CAR: forward primer GGCCTCAGTGAAGATGTCCT, reverse primer TAGGCTGTGCTGGAGGATTT). The total amount of genomic DNA was used as the internal control for quantitation. The CAR expression plasmid was used as the internal reference. PCR detection was performed using a LightCycler 480II system (Roche). According to qPCR, the corresponding Cq (STD0) -Cq (STDn) was set as X, Log10 (Copy # STD) was set as Y, and a standard curve was obtained. Through the standard curve and the corresponding sample X value Cq (control) -Cq (sample), the Y value was obtained, and the operation 10^y^ was conducted, revealing the copy/100 ng genomic DNA.

*Image analysis.*

The PET-CT scan was used to evaluate the tumor burden before treatment and the efficacy of CAR-T cell therapy. A Discovery PET/CT Elite (GE) system was used for PET-CT. All patients were fasted for more than 6 h prior to the scan and had no history of a barium meal or barium enema for the previous 7 days. The fasting blood glucose of patients was within the normal range. 18F-FDG at 0.1mCi/kg (body weight) was injected intravenously and the patients rested 60 minutes before the scanning from the top of the head to the proximal thigh, with a scan layer thickness of 3.75mm and a slice interval of 3.27mm. The reconstruction algorithm was filtered back projection (FBP). SUV was the tissue radioactive concentration (KBq/ml) divided by the injection dose per unit weight (KBq/g). SUVmax was measured using the 3D measurement method. A volume of interest (VOI) was placed on each PET/CT fusion image to cover the entire tumor volume. The threshold of volume with SUVmax was 40% of the SUVmax to determine the tumor margin, the tumor metabolic volume (TMV) and mean SUV (SUVmean). TLG = SUVmean × MTV.

*Measurement of serum cytokines.*

The concentrations of serum interleukin-6 (IL-6), interferon (IFN)-γ and tumor necrosis factor α (TNF-α) were determined using enzyme-linked immunosorbent assay (ELISA), according to the manufacturer’s instructions (R & D systems, Minneapolis, USA).
